# Evaluation of the changes in incidence and patient age of knee arthroscopy along with changes in time between knee arthroscopy and arthroplasty between 1998 and 2018: a nationwide register study

**DOI:** 10.1186/s43019-023-00194-2

**Published:** 2023-07-11

**Authors:** Ville T. Ponkilainen, Mikko Uimonen, Raine Sihvonen, Nikke Partio, Juha Paloneva, Ville M. Mattila

**Affiliations:** 1 Department of Surgery, Hospital Nova, Central Finland Healthcare District, Hoitajantie 3, 40620 Jyväskylä, Finland; 2 Pihlajalinna Koskisairaala Hospital, Tampere, Finland; 3 Department of Orthopaedics and Traumatology, Tampere University Hospital, Teiskontie 35, PL2000, 33521 Tampere, Finland; 4 Faculty of Health Sciences, University of Eastern Finland, Kuopio, Finland; 5 Faculty of Medicine and Health Technology, Tampere University, Tampere, Finland; 6 COXA Hospital for Joint Replacement, Biokatu 6, 33520 Tampere, Finland

## Abstract

**Background:**

Recent evidence has led to guidelines to refrain from recommending knee arthroscopy for patients with an osteoarthritis diagnosis. The aim of this study was to evaluate the latest changes in the incidence of arthroscopic surgery for degenerative knee disease, changes in the ages of those patients and the delay between knee arthroscopy and arthroplasty, in Finland between 1998 and 2018.

**Method:**

The data for were collected from the Finnish National Hospital Discharge Register (NHDR). All knee arthroplasties and arthroscopies performed due to osteoarthritis, degenerative meniscal tears, and traumatic meniscal tears were included. Incidence rates (per 100,000 person-years) as well as the median age of patients were calculated.

**Results:**

The incidence of arthroscopy decreased 74% (413 to 106 per 100,000 person-years) and knee arthroplasty increased 179% (94 to 262 per 100,000 person-years) between 1998 and 2018. The incidence of all arthroscopies increased until 2006**.** Subsequently, the incidence of arthroscopy due to OA decreased by 91% and arthroscopic partial meniscectomy (APM) for degenerative meniscal tears decreased by 77% until 2018. The decrease of traumatic meniscal tears begun later, leading to decrease of 57% between 2011 and 2018. Conversely, the incidence of patients undergoing APM of traumatic meniscal tear increased 375%. The median age of patients who underwent knee arthroscopy decreased from 51 to 46 and from 71 to 69 in knee arthroplasty patients.

**Conclusions:**

Increasing evidence that recommends refraining from knee arthroscopy in OA and degenerative meniscal tears has led to a dramatic decrease in the incidence of arthroscopies. Simultaneously, the median age of the patients who undergo these operations has continued to decrease.

**Supplementary Information:**

The online version contains supplementary material available at 10.1186/s43019-023-00194-2.

## Introduction

In 2002, a placebo-controlled surgical trial published by Moseley et al. concluded that arthroscopic knee debridement or lavage does not improve outcome when compared to placebo procedure [1–3]. This conclusion was subsequently supported by the findings of a trial by Kirkley et al. in 2008 [4]. This new evidence led to guidelines and expert opinion that refrained from recommending knee arthroscopy for patients with an established osteoarthritis (OA) diagnosis [5, 6]. However, for those patients with torn meniscus or intra-articular loose fragments with low-grade osteoarthritis, knee arthroscopy was still recommended [5–7]. Consequently, the rates of knee arthroscopy for knee osteoarthritis started to decrease [8, 9].

As patients with degenerative knee disease should be treated conservatively and knee arthroscopy should be carefully used only in younger patients, the mean age of patients undergoing knee arthroscopy should be decreasing. However, the recommendations to refrain from performing arthroscopy on degenerative knees should not have an influence on the number or the mean age of patients undergoing arthroscopic meniscus repair, as it is still the preferred treatment option for acute traumatic meniscal tears, especially in the young active population. [10, 11]

The aim of this study was to evaluate the latest changes in the incidence of arthroscopic surgery for degenerative knee disease, changes in the ages of those patients and the delay between knee arthroscopy and arthroplasty, in Finland between 1998 and 2018.

## Materials and methods

The data for this retrospective nationwide register study were collected from the Finnish National Hospital Discharge Register (NHDR) from January 1st, 1998, through December 31st, 2018. Data on age, sex, hospital (public vs. private), primary and secondary operation codes, and diagnosis were obtained from the register. The coverage and accuracy of the NHDR have been shown to be excellent [12–14].

In Finland, the healthcare system is divided into public health care, which is accessible to all permanent residents of Finland and into private sector, which is also accessible to everyone, but the costs are covered by the patients themselves or by health or accident insurance or by private occupational health care bodies. Our data contains all operations performed in both public and private hospitals.

In this study, the Finnish procedural codes maintained by The Nordic Medico-Statistical Committee (NOMESCO) [15] were used for procedural codes, and the ICD-10 coding system was used for diagnoses. In the NHDR, the procedure codes and diagnoses are recorded by the operating surgeon, and therefore multiple codes may be recorded. Knee operations related to OA and meniscal tear were included and categorized based on the ICD-10 code. The diagnosis code was subsequently combined with the procedural codes and formed the following four groups: (I) Arthroscopy due to osteoarthritis, (II) Arthroscopic partial meniscectomy (APM) of degenerative meniscal tear, (III) APM of traumatic meniscal tear, (IV) Repair of traumatic meniscal tear, and (V) Primary knee arthroplasty (Additional file 1: Appendix S1). Both unicompartmental and total knee arthroplasties (TKA) were included.

Patients with knee OA were included using the codes M17* (OA). The codes M23.2 and M23.3 were used for degenerative meniscal tears, and the code S83.2 (acute meniscal tear) was used for tears considered traumatic by the operating surgeon. In cases of multiple applicable knee diagnoses for the same surgical procedure, the procedure was categorized according to the more degenerative diagnosis (Osteoarthritis > Degenerative meniscal tear > Traumatic meniscal tear).

The data for recurrent arthroscopies and knee arthroplasty were analyzed patient-wise. (1) Recurrent knee arthroscopies were evaluated by including all arthroscopies for each patient, and by calculating the time difference between the first and the second knee arthroscopy during this period. The recurrent arthroscopies were presented after one and three years of follow-up. (2) The time difference between arthroscopy and arthroplasty was calculated from the time difference from the last arthroscopy to the first knee arthroplasty. All arthroscopies performed 10 years prior to the first knee arthroplasty were included. The primary operations were limited so that they began from 2008, ensuring a similar follow-up period for each patient. For reoperations and previous operations before TKA, the same inclusion criteria as for primary surgeries was used.

### Statistical analysis

The annual incidences (per 100 ,000 person-years) were calculated based on the entire adult population (age ≥ 18 years) of Finland obtained from the national population register (Official Statistics of Finland). The annual mean of the population, comprising approximately 4 500,000 citizens in 1998 and 4,900,000 citizens in 2018, was used. All incidences were weighted by age and gender. The continuous variables were presented as mean with standard deviation (SD) or as median with interquartile range (IQR) based on the distribution of the variable. The normality of the continuous variables was confirmed by inspection of histograms for the corresponding groups. All analyses were performed using R version 4.0.5 (R Foundation for Statistical Computing, Vienna, Austria).

### Ethical approval

According to Finnish research legislation and the Finnish National Board on Research Integrity, appointed by the Ministry of Education and Culture: "The review of the ethics committee is not required for the research of public and published data, registry and documentary data and archive data.". Thus, ethical approval was not required. However, the institutional permit was obtained from the Finnish Institute for Health and Welfare (Permission number: THL/1800/5.05.00/2019).

## Results

**Fig. 1:**
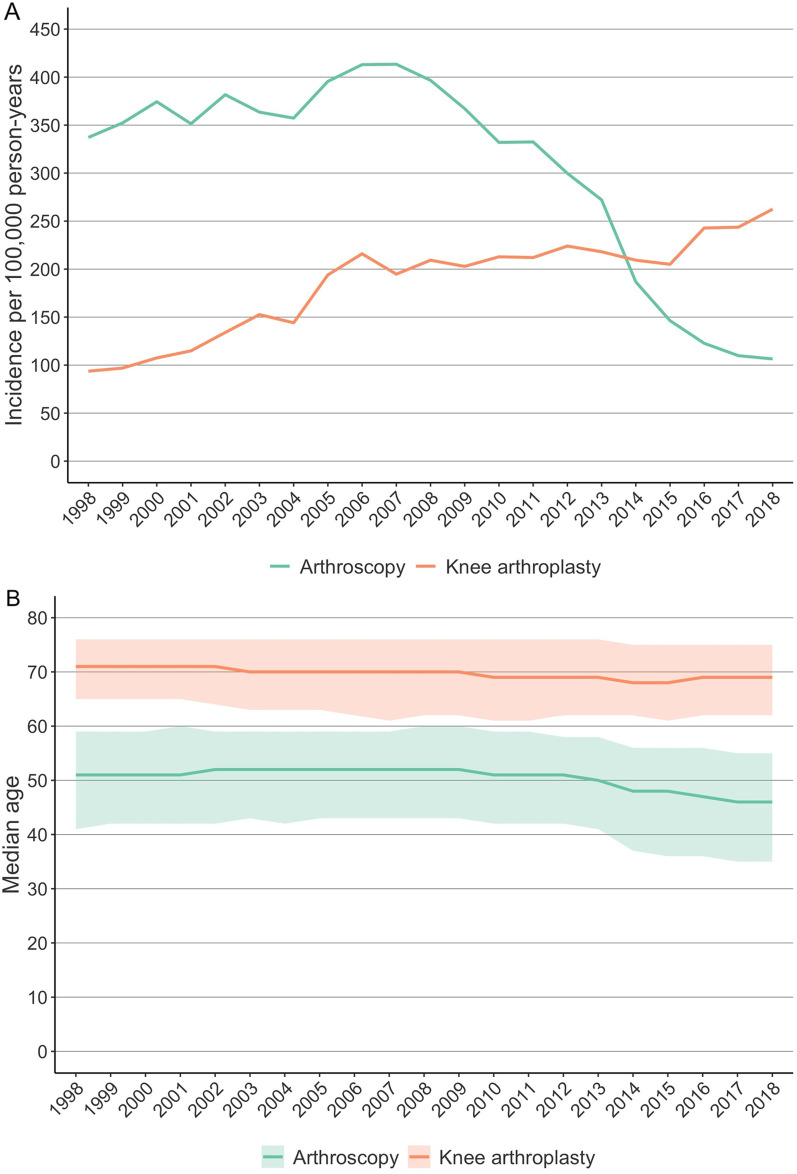
The incidence **A** and the median age (IQR) **B** of patients undergoing arthroscopic knee operations due to meniscal tears, degenerative meniscal tears, or osteoarthritis in Finland between 1998 and 2

The data comprised a total of 299 326 knee arthroscopies and 184 473 arthroplasties. The incidence of arthroscopic knee procedures decreased by 74% from 413 per 100,000 person-years in 2007 to 106 per 100 000 person-years in 2018 (Fig. 1A). In addition, the incidence of knee arthroplasty increased by 179% from 94 per 100 000 person-years in 1998 to 262 per 100 000 person-years in 2018. During the same period, the median (IQR) age of the patients who underwent knee arthroscopy decreased from 51 (41 to 59) to 46 (35 to 55), and the median (IQR) age of knee arthroplasty patients decreased from 71 (65 to 76) to 69 (62 to 75) (Fig. 1B).

**Fig. 2:**
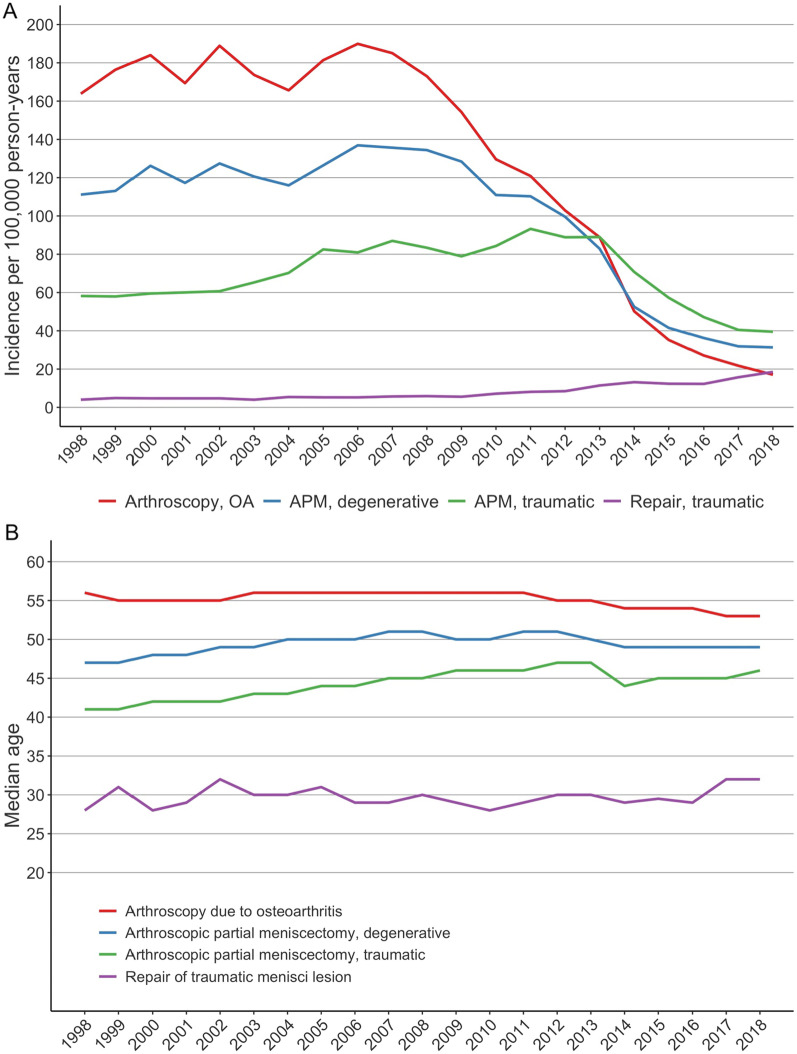
The incidence of knee arthroscopy **A** and the median age **B** of patients undergoing arthroscopic treatment in Finland between 1998 and 2018

Initially, the incidence of all arthroscopies increased between 1998 and 2006 (Fig. 2A), but subsequently began to decrease. Indeed, between 2006 and 2018, arthroscopy due to OA decreased by 91% and APM for degenerative meniscal tears decreased by 77%. Furthermore, the incidence of arthroscopies for traumatic meniscal tears decreased by 57% between 2011 and 2018. For meniscal repairs, the incidence increased continuously from 4 to 19 per 100 000 person-years between 1998 and 2018, resulting in an increase of 375%.

The median age for all arthroscopy patients slowly increased between 1998 and 2013 and then decreased rapidly between 2013 and 2014 (Fig. 2B). Between 2011 and 2018, the median age of patients undergoing arthroscopy for OA decreased from 56 to 53 years, whereas for those patients undergoing APM of degenerative meniscal tear median age increased from 47 to 49 years. The median age of patients undergoing APM of traumatic meniscal tear increased from 41 to 46 years. The median age of patients undergoing meniscal repair increased continuously from 28 to 32 years.

**Fig. 3:**
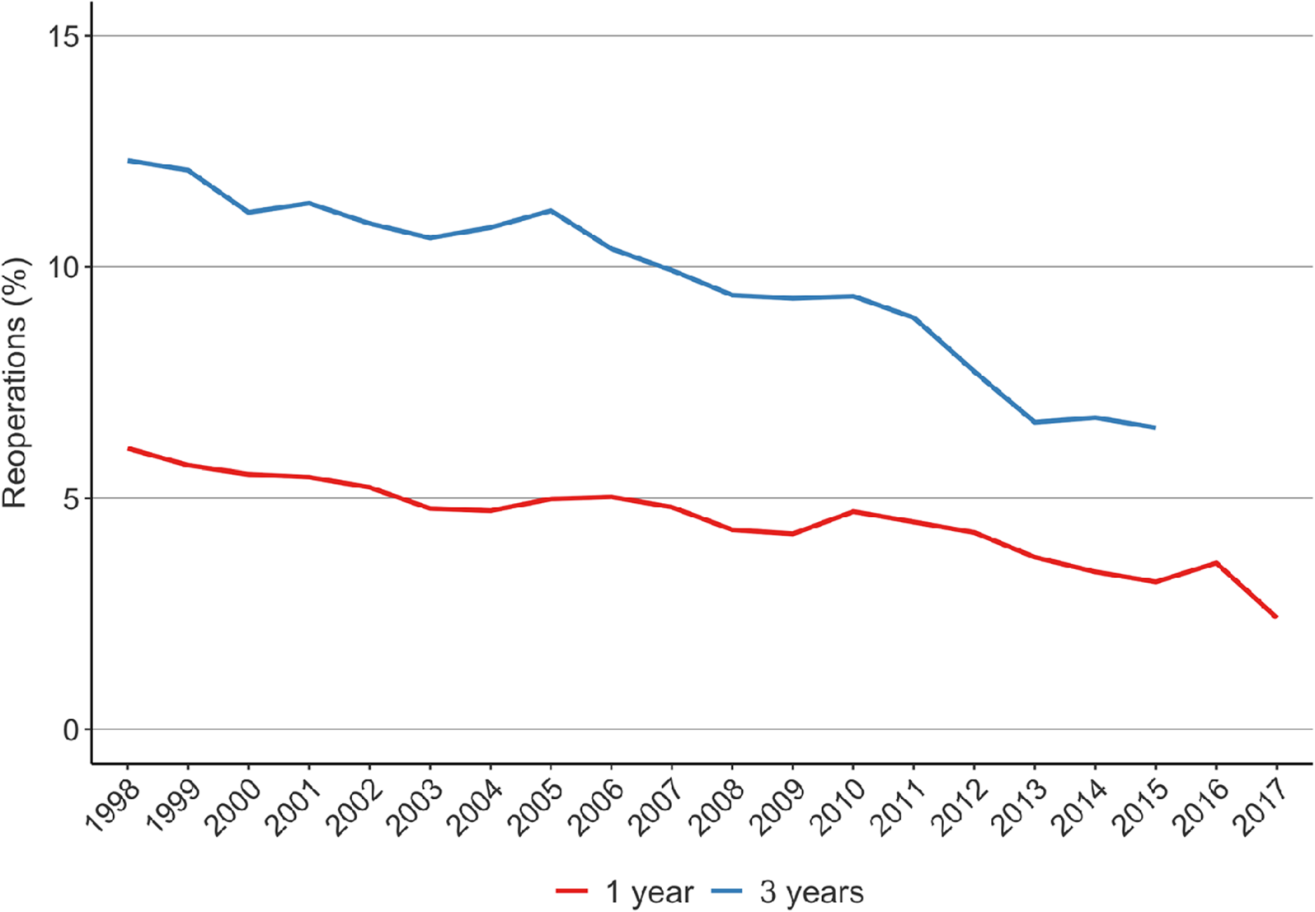
The rate (%) of recurrent arthroscopies after primary knee arthroscopy per patient after 1 and 3 years of follow-up in Finland between 1998 and 2017

**Fig. 4:**
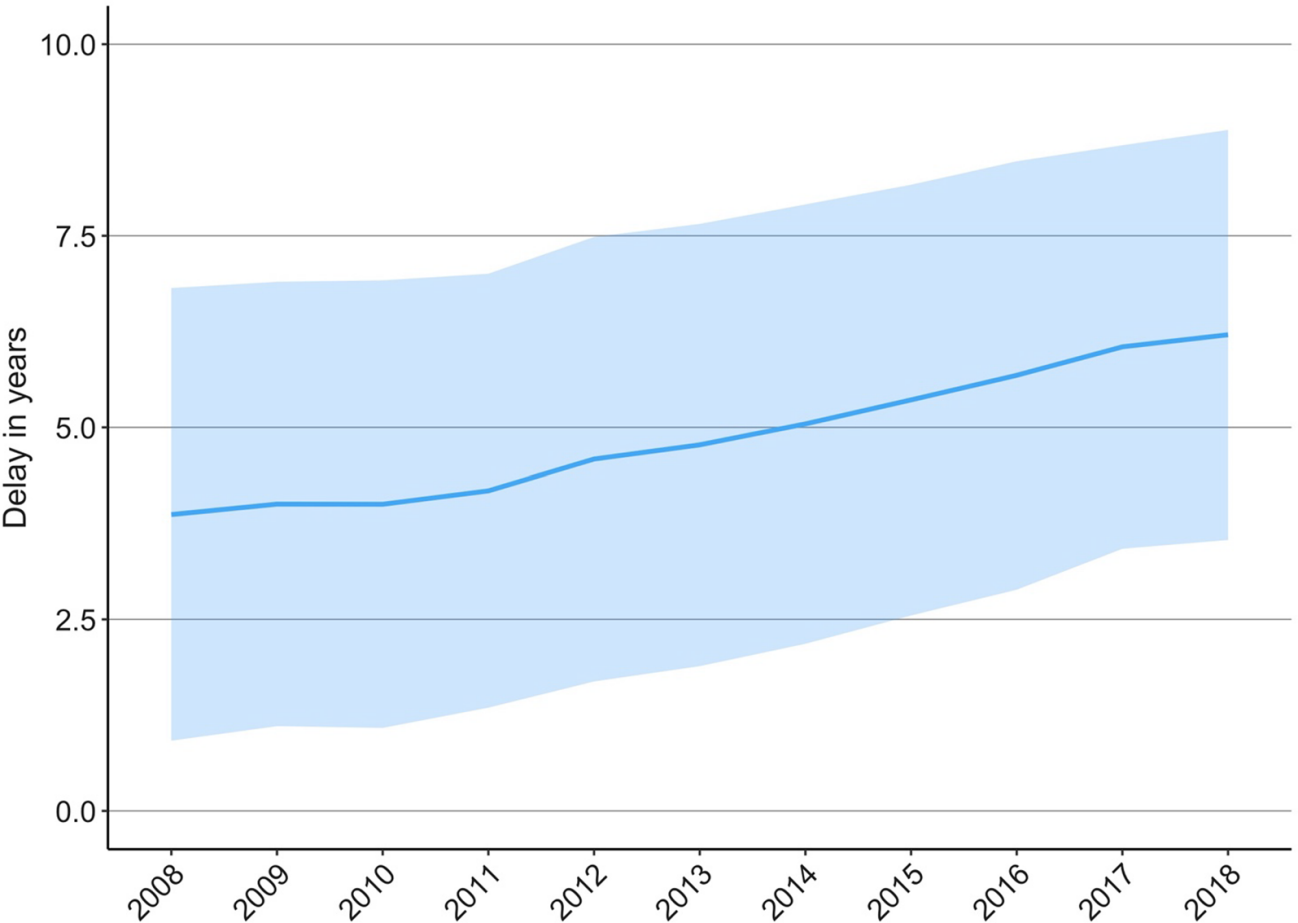
The mean (+ -SD) duration between operations in patients who underwent arthroscopy 10 years before the first knee arthroplasty in Finland between 2008 and 2018

Between 1998 and 2017, the percentage of recurrent knee arthroscopies 1 year after primary arthroscopy decreased from 6.1% to 2.4% (Fig. 3). Similarly, between 1998 and 2015, the percentage of recurrent knee arthroscopies 3 years after primary arthroscopy decreased from 12.3% to 6.5%. In addition, the mean delay between knee arthroscopy and arthroplasty increased from 3.9 (SD 3) years to 6.2 (SD 2.7) years between 2008 and 2018 (Fig. 4).

## Discussion

The main finding of the present study is that the incidence of degenerative knee arthroscopies is rapidly decreasing, indicating that the high-quality evidence has had clear impact on the clinical practices regarding the arthroscopic treatment of knee osteoarthritis and degenerative meniscal tears. The decrease first occurred in 2006 among patients undergoing arthroscopy for OA and arthroscopic partial meniscectomy for degenerative and traumatic meniscal tears. The decrease became more pronounced after 2013. Conversely, the incidence of menisci repairs increased continuously between 1998 and 2018. The recurrent arthroscopy rate after the first knee arthroscopy decreased by more than 50% between 1998 and 2017, and the delay between arthroscopy and arthroplasty increased from 4 to 6 years. Moreover, during the same period, the incidence of knee arthroplasty almost tripled, and the median age of patients decreased from 71 to 69 years.

The finding of a decreasing incidence of arthroscopies is likely a result from the implementation of evidence-based medicine in surgery and its dissemination in Finland. The placebo-controlled study by Moseley et al. (2002) was the first high quality trial to report that knee arthroscopy for patients with osteoarthritis did not improve outcome [1]. The number of such arthroscopies decreased quite rapidly after the publication of the study findings (approximately 2006), four years after the highest peak in knee arthroscopy for degenerative knee disease and osteoarthritis was recorded. Thereafter, these trends remained stable, but another steep decrease occurred after 2013. This decrease was probably related to the findings of the placebo-controlled trial of arthroscopic partial meniscectomy for degenerative meniscus tears by Sihvonen et al. [2] that was conducted in Finland. Due to the local nature of the trial, the study received more media attention in Finland than the study by Moseley et al. Consequently, it affected the policies of the insurance companies, which discontinued compensation for surgery for degenerative meniscus.

In an earlier Finnish epidemiological study by Mattila et al. [8], the onset of a decreasing trend in degenerative knee arthroscopy had already been noted. In their study, the data were limited to the year 2012, when the most pronounced decrease had not yet been reached. More interestingly, the mean age of the patients had not at that time turned into a clear decrease. Nevertheless, the mean age of the patients began to decrease prominently after 2013. This may be seen as the result of the emerging evidence on degenerative knee disease. In addition, more strict insurance compensation policies may favor the use of a traumatic tear code in the treatment of degenerative treat rather than a non-traumatic code. As hypothesized, however, the decreasing mean age in all diagnosis groups, except in the traumatic menisci lesions group, indicates that the research-based evidence has been well adopted.

In the present study, a continuous increase in the incidence of menisci repairs were observed. Against the expectations, the median age of this patient group simultaneously increased. Of all the arthroscopic operations for meniscus-related diseases, an arthroscopy for traumatic lesions is the only one that is still recommended in the literature [10]. In our nationwide sample, the median age of patients was 32 (IQR 25 to 41) years, which means that 50% of these patients are aged between 25 and 41. While it is difficult to distinguish those patients with truly traumatic lesions and those with degenerative lesions [16–19], increasing mean age may indicate that there exists a meaningful proportion of degenerative meniscus lesions in this group that are labeled as traumatic ones. It is also possible that in patients undergoing MRI after a minor injury, the existent of a degenerative meniscal tear might be considered to be a result of the injury, although the proportion of incidental meniscus lesions has been found to be considerable. [19] Possible reasons behind the increasing incidence of meniscus repairs is that it has been reported to result in higher functional scores and less radiologic degeneration than APM in the long term [20]. A second possible factor is that the techniques for the repair have advanced during recent years [21]. These changes have led to a drift away from resection towards repair, known in the literature as the: “*Save the meniscus”* campaign [22, 23].

Knee arthroplasty is the definitive treatment of end-stage knee OA [24]. However, younger patients are at higher risk for perceiving more knee-related dysfunction and dissatisfaction in addition to early periprosthetic joint infection and aseptic mechanical failure leading to revision surgery. Therefore, the operation should be delayed for as long as possible. [25–27] The increasing incidence of knee arthroplasty has been previously reported [28]. Furthermore, it has also been reported that the incidence of knee arthroplasty in Finland is the highest among the Nordic countries. Data from the Nordic arthroplasty register showed that in 2012 the incidence of knee arthroplasty was 280 in Finland, 210 in Sweden and Denmark, and 140 in Norway per 100 000 inhabitants. [28] The reasons for the higher incidence in Finland have been hypothesized previously by Niemeläinen et al. [28] One of the hypotheses, especially for the rapid rise that occurred between 2004 and 2006, is that new social and health care regulations obliged hospitals to shorten patient waiting times for surgery.

For comparison, the mean age of all knee arthroplasty patients in Sweden decreased from 71 to 69, [29] between 1995 and 2018, and therefore strictly followed the same age trend we have reported in the present study from Finland. The suggested reasons behind the increasing incidence of younger knee arthroplasty patients may be the increasing prevalence of obesity [30, 31] and the popularity of contact sports, [32] leading to a higher prevalence of knee OA in younger patients. In addition, the development of fast-track surgery may have made the process easier for younger patients and also increased the volume of arthroplasty hospitals [33, 34].

Previously, knee arthroscopy was regarded as a pre-stage option for patients with degenerative knee disease, and it was often performed more than once prior to knee arthroplasty [8]. In the present study, a 61% increase in the mean duration between knee arthroscopy and the first knee arthroplasty were found, reaching a mean duration of 6.2 years before the knee arthroplasties performed in 2018. Although the change has been rapid and is in line with corresponding duration in other countries, it is still notably shorter than that reported in Italy (2015, 13.3 years in 20 years of follow-up) and the USA (2011, 9 years in 10 years of follow-up). [35] It should be noted, however, that these previous studies did not include nationwide trends, and therefore include more uncertainty. In addition, many of the studies investigating the delays have had a follow-up of only 1 to 3 years, and thus we were unable to perform direct comparisons [35].

The main strength of the study is the representative data including all operations performed in all hospitals in Finland between 1998 and 2018. In addition, the data reliably included the age of all patients, and we were therefore able to investigate the true changes in the mean age. The main weakness is that the study groups are based on the diagnosis and operation codes determined by the operating surgeon, and traumatic APM group may contain both erroneous traumatic diagnosis codes representing non-traumatic meniscal tears. In addition, there might be variation between the codes used by individual surgeons. Secondly, the data represents Finnish health care and is not generalizable to other nations and health care systems.

In conclusion, increasing evidence that refrains from recommending knee arthroscopy for OA and degenerative meniscal tears has led to a dramatic decrease in the incidence of these operations. Simultaneously, the median age of the patients undergoing these procedures has continuously decreased.

## Supplementary Information

**Additional file 1.** The classification for arthroscopic knee operations

## Data Availability

Due to permission policies, the authors are not allowed to share the data used in this article.

## Declarations

### Patient involvement

Patients were not involved in the planning or conducting of this study.
