# Supplementary material for: Evaluation of the changes in incidence and patient age of knee arthroscopy along with changes in time between knee arthroscopy and arthroplasty between 1998 and 2018: a nationwide register study
Source: Knee Surg Relat Res. 2023 Jul 11;35:19. doi: 10.1186/s43019-023-00194-2 (PMC10334544; doi:10.1186/s43019-023-00194-2)
Supplement: Supplementary file 1 — Additional file 1. The classification for arthroscopic knee operations [file 43019_2023_194_MOESM1_ESM.docx]

Additional file 1: Appendix S1. The classification for arthroscopic knee operations.

| I Arthroscopy due to osteoarthritis |  |
| --- | --- |
| M17.0 Primary knee osteoarthritis, bilateral | NGA30 Arthroscopic exploration of knee joint |
| M17.1 Gonarthrosis, bilateral | NGD05 Arthroscopic partial resection of menisci |
| M17.3 Other post-traumatic gonarthrosis | NGF25 Arthroscopic resection of knee joint |
| M17.9 Gonarthrosis, unspecified | NGF35 Arthroscopic partial excision of joint cartilage of knee |
|  |  |
| II Arthroscopic partial meniscectomy of degenerative meniscal tear |  |
| M23.2 Derangement of meniscus due to old tear or injury | NGA30 Arthroscopic exploration of knee joint |
| M23.3 Other meniscal derangements | NGD05 Arthroscopic partial resection of menisci |
|  | NGF25 Arthroscopic resection of knee joint |
|  | NGF35 Arthroscopic partial excision of joint cartilage of knee |
|  |  |
| III Arthroscopic partial meniscectomy of traumatic meniscal tear |  |
| S83.2 Tear of meniscus, current | NGA30 Arthroscopic exploration of knee joint |
|  | NGD05 Arthroscopic partial resection of menisci |
|  | NGF25 Arthroscopic resection of knee joint |
|  | NGF35 Arthroscopic partial excision of joint cartilage of knee |
|  |  |
| IV Repair of traumatic meniscal tear |  |
| S83.2 Tear of meniscus, current | NDG25 reinsertion of meniscus |
|  |  |
| V Primary knee arthroplasty |  |
| All diagnoses above | NGB10 Primary partial prosthetic replacement of knee joint, demi prosthesis |
|  | NGB20 Primary total prosthetic replacement of knee joint without patellar part |
|  | NGB30 Primary total prosthetic replacement of knee joint without patellar part - connected prosthesis |
|  | NGB40 Primary total prosthetic replacement of knee joint with patellar part - sliding prosthesis |
|  | NGB50 Primary total prosthetic replacement of knee joint with patellar part - connected prosthesis |
